# Epigenetic polypharmacology: from combination therapy to multitargeted drugs

**DOI:** 10.1186/s13148-016-0271-9

**Published:** 2016-10-12

**Authors:** Angel R. de Lera, A. Ganesan

**Affiliations:** 1 Departamento de Química Orgánica, Facultade de Química, Universidade de Vigo, CINBIO and IIS Galicia Sur, 36310 Vigo, Spain; 2 School of Pharmacy, University of East Anglia, Norwich Research Park, Norwich, NR4 7TJ UK

**Keywords:** Polypharmacology, Epigenetic drugs, Combination therapies

## Abstract

The modern drug discovery process has largely focused its attention in the so-called magic bullets, single chemical entities that exhibit high selectivity and potency for a particular target. This approach was based on the assumption that the deregulation of a protein was causally linked to a disease state, and the pharmacological intervention through inhibition of the deregulated target was able to restore normal cell function. However, the use of cocktails or multicomponent drugs to address several targets simultaneously is also popular to treat multifactorial diseases such as cancer and neurological disorders. We review the state of the art with such combinations that have an epigenetic target as one of their mechanisms of action. Epigenetic drug discovery is a rapidly advancing field, and drugs targeting epigenetic enzymes are in the clinic for the treatment of hematological cancers. Approved and experimental epigenetic drugs are undergoing clinical trials in combination with other therapeutic agents via fused or linked pharmacophores in order to benefit from synergistic effects of polypharmacology. In addition, ligands are being discovered which, as single chemical entities, are able to modulate multiple epigenetic targets simultaneously (multitarget epigenetic drugs). These multiple ligands should in principle have a lower risk of drug-drug interactions and drug resistance compared to cocktails or multicomponent drugs. This new generation may rival the so-called magic bullets in the treatment of diseases that arise as a consequence of the deregulation of multiple signaling pathways provided the challenge of optimization of the activities shown by the pharmacophores with the different targets is addressed.

## Background

### Principles of polypharmacology

Notwithstanding the success of combination therapy, the use of a single drug that modulates several targets might be therapeutically advantageous over the use of drugs in combination. In cancer, the design and synthesis of new molecules that simultaneously modulate multiple biochemically distinct oncogenic targets is of current interest. Polypharmacology refers to the ability of drugs to interact simultaneously and specifically with multiple targets (multitarget drugs). Although polypharmacology might be associated with compound promiscuity, it should be considered a different category since the so-called promiscuous drugs are instead a class of compound that show a wide spectrum of biological activities and adverse reactions [1].

Among the advantages of multitarget drugs vs drug combinations are the more predictable pharmacokinetic (PK) and pharmacodynamic (PD) relationship of the components of a single medicine, the possibility that one motif might improve the bioavailability of the second entity, the greater efficacy against advanced-stage diseases, the lower toxicities, the simultaneous presence of the chemical entities in multiple tissues, and the improved patient compliance [2]. To benefit from those effects, it is required that the multitarget drug exhibit balanced in vitro and in vivo activities to match potency for the corresponding targets, as well as optimized PK and safety profiles. A combination of drugs faces the problem of the different solubilities that may modify the bloodstream uptake, which requires fine-tuning the formulation in order to ensure the required blood level of each drug. In addition, the regulatory requirements are more complex when the agents are used in combination, since the safety profile of each drug needs to be demonstrated before clinical trials, and this can be further delayed due to regulatory and IP issues, in particular if the two drugs are being developed by different companies [1].

Efforts are underway to use chemoinformatics to help understand drug effects from a signal transduction network perspective [3], to confidently predict new molecular targets for known drugs, and to explain polypharmacology. Another current trend in therapy is drug repurposing or the re-discovery of a new therapeutic area for a drug used traditionally to treat a given pathology, either through the ability to modulate an additional target or by the involvement of the primary target in multiple pathologies. Examples include the use of the anti-angina drug sildenafil to treat sexual disfunction or the infamous sedative thalidomide as therapy for multiple myeloma. Indeed, the polypharmacology of current drugs has been studied using a statistical ligand-based approach [4]. This study, aimed to discover chemical similarities between drugs and ligand sets, has revealed unanticipated promiscuities but also previously unreported polypharmacologies. The screening study of the 3665 FDA-approved and investigational drugs was conducted using databases containing the chemical structures of hundreds of thousands of biologically active compounds for which the binding characteristics to a panel of 1400 target proteins were known [4]. A massive network of interactions (nearly 7000 of them with high probability) for the studied compounds with off-targets were predicted, which indicates that polypharmacology is, perhaps unintentionally, a feature intrinsic to the therapeutic efficacies of drugs.

Multikinase inhibitors, for example, initially considered to be highly specific for one of the 518 kinases of the kinome, have proven successful in treating previously refractory cancers, perhaps as a result of simultaneous inhibition of multiple kinases. As an example, sunitinib, a promising drug for the treatment of anaplastic thyroid cancer, inhibits 79 kinases with *K*_D_ < 10 μM. Therefore, the success of (multi)kinase inhibitors in treating cancer is a consequence of the modulation of multiple signaling pathways that support cancer cell proliferation, apoptosis, angiogenesis and recruitment of surrounding tissues.

Also, in infectious diseases, current drugs show off-target effects. This is the case with the HIV protease inhibitor nelfinavir, which has been found to also inhibit the proliferation of cancer cells due to a weak modulation of multiple kinases.

Polypharmacology is prevalent in the area of CNS diseases. The activity of drugs acting on the CNS is often mediated by G protein-coupled receptors (GPCRs), a group of receptors that are also implicated in multiple therapeutic areas and share structural and functional similarities that makes selectivity a very difficult issue. For example, the use of atypical antipsychotic clozapine is associated with undesired side effects, such as diabetes and seizures, which may be due to its broad range of targets, among them different isoforms of the serotonin, dopamine, muscarinic, and adrenergic receptors, members of the GPCR superfamily. On the other hand, a single drug exhibiting polypharmacology for more than one target of the same disease could exhibit synergistic effects. This is the case of ladostigil, an inhibitor of acetylcholine esterase (AChE) and the brain monoamine oxidases (MAO) A and B, which has shown efficacy in models of Alzheimer’s disease.

**Scheme 1:**
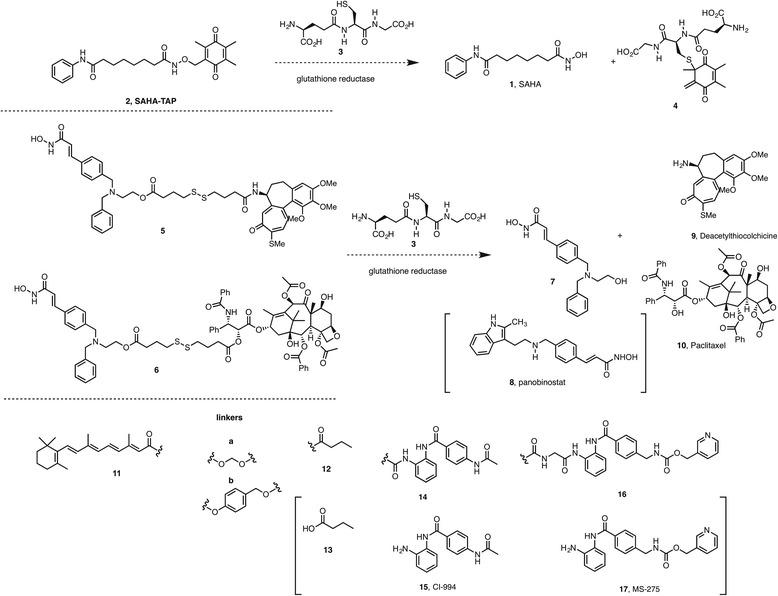
Examples of pro-drugs and mutual pro-drugs containing an HDACi and release mechanisms. In brackets, the structures of the corresponding HDACis

Multitarget drugs exhibiting polypharmacology due to their ability to modulate as single chemical entities multiple targets simultaneously are also termed multiple ligands [5] and hybrid molecules [6]. These molecules should not be considered as pro-drugs, which are those designed to correct the pharmacokinetic and pharmacodynamic profiles of a valuable lead. For example, the hydroxamic acid functionality of the approved histone deacetylase inhibitor (HDACi) vorinostat (also known as SAHA, suberoylanilide hydroxamic acid, **1**) was covalently bound to a thiol-sensitive group in the design of a dual-mode HDAC prodrug (SAHA-TAP, **2**) in order to facilitate the delivery of the drug, which itself has poor pharmacokinetics [7]. Selective activation by glutathione **3**, which is present at higher concentrations in cancer cells (1 mM) than in the intracellular compartment (1 μM), would release the hydroxamate of **1** upon conjugate addition to the quinone giving **4** (Scheme 1).

Hybrid molecules [6], in contrast to pro-drugs, contain two (or more than two) domains with different biological functions and dual activities that ideally act as distinct pharmacophores, although not necessarily on the same biological target. Thus, multiple ligands usually consist of the combination of pharmacophores of selective ligands (either already known drugs or candidates). From the point of view of the medicinal chemist, pharmacophores that are similar and share common substructures, usually hydrophobic or basic ring systems, can be synthetically fused or merged (see examples in next section). Alternatively, if pharmacophores are dissimilar, they can be joined as conjugates with cleavable or non-cleavable linkers, although this strategy often leads to structures of high molecular weight (MW) and lipophilicity [5].

When both pharmacophores are connected by a linker that is labile or can be easily cleaved in vivo, they are called dual or mutual pro-drugs since each of them uses another pharmacologically active compound instead of some inert molecule as carrier. Being released simultaneously inside the cancer cells, they might act synergistically and affect distinctive cellular targets, in contrast to the simultaneous administration of two individual synergistic agents, which are usually transported to the site of action with different efficiencies.

Examples of hybrid anticancer molecules containing an epi-drug and another antitumor agent connected via a linker are shown in Scheme 1. The scaffold of the HDACi dacinostat (LAQ-824, compound **7**; an early candidate that was further improved as panobinostat **8**) and a tubulin binder (thiocolchicine **9** and paclitaxel **10**) were connected via a disulfide bond as in **5** and **6** [8]. Glutathione **3** would release the thiolates via disulfide exchange reactions, which in turn would produce the thiolactones to free the second component.

Mutual pro-drugs of all-*trans*-retinoic acid (ATRA) and several HDACis (butyric acid **13**, tacedinaline **15** and entinostat **17**) have been engineered via glycine acyloxyalkyl carbamate linker (which would be presumably cleaved by esterases, compounds **11a**-**12** and **11a**-**14**) or through a benzyl ester linker (which would be presumably released through a 1,6-elimination reaction, compounds **11b**-**12** and **11b**-**16** and **11b**-**14**) [9]. The last series of mutual pro-drugs showed potent inhibition of the growth of several hormone-insensitive/drug resistant breast cancer cell lines and the hormone-insensitive PC-3 prostate cancer cell line [10].

Still, the development of multitarget drugs from leads is more complex than that of single drugs. Drug-like molecular properties for multiple pharmacological activities must be optimized and unintended interactions with additional targets minimized. Moreover, balancing the pharmacological activities is another complication, as often the optimal ratio is not 1:1. For example, although the hybrid compounds **5** and **6** were able to retain antimitotic and proapoptotic activity, the potency of the construct was lower than anticipated [8].

Finally, according to the mechanism of action, hybrid molecules can be classified in three different categories: (a) both entities interact with the same target (“double sword” molecules); (b) both entities independently interact with two different and nonrelated targets; (c) both entities interact simultaneously with two related targets at the same time [6].

## Main text

### Challenges for rational epigenetic drug polypharmacology

The new paradigm of single chemical entities that antagonize multiple biochemically distinct targets to overcome conventional single-target therapeutics is being pursued in the epigenetic field, in particular for the treatment of cancer [11, 12]. The challenge in this field is the design of small molecules that have the property to modulate at the same time several of the epigenetic targets with contrasting or totally unrelated mechanism of action. Promiscuity, traditionally considered an undesired property of drugs, might turn out to be advantageous also in epigenetics and the polypharmacology of these epi-drugs a feature intrinsic to their therapeutic efficacies.

In principle, since some of the epigenetic enzymes such as sirtuins (SIRTs), protein arginine methyltransferases (PRMTs), DNA methyltransferases (DNMTs), and lysine methyltransferases (KMTs) use the same cofactor or cofactors containing adenosine, modulators of several of these enzymes that bind to the corresponding adenosine pockets can be designed, and moreover, these might also cross-react with related receptors such as kinases. Likewise, the metalloenzymes HDACs and Jumonji lysine demethylases (KDMs) can be subjected to simultaneous inhibition with metal-chelating containing compounds. However, these simple assumptions cannot be extrapolated to the different protein families. For example, the *S*-adenosyl methionine (SAM) binding site of lysine methyltransferases is more extended than that of other enzymes using the same cofactor such as DNMTs. Both the SAM cofactor and the substrate of KMTs access the protein from opposite faces in domains linked through a narrow hydrophobic channel. In addition, the SAM cofactor adopts different conformations in the domains of KMTs compared to PRMTs although both enzymes transfer a methyl group to protein side-chains.

Even more challenging is the inhibition of epigenetic enzymes with unrelated mechanistic principles. To get a glimpse of the difficulties expected in the rational design of multiple epigenetic ligands, a brief description of the reaction mechanisms for the most common epigenetic enzymes follows.

#### Writers/erasers of acetyl groups

The acetylation status of lysine **ε**-amino residues in histones is under the control of the opposing activities of histone deacetylases and histone acetyltransferases (HDACs and HATs, respectively). In addition to the regulation of chromatin function and structure, acetylation has a broad regulatory role in many biological processes (cell cycle, splicing, nuclear transport, actin nucleation [13], cellular metabolism [14, 15], etc.) beyond chromatin remodeling. These effects might be due to the modulation by the acetylation/deacetylation mechanistic switch of the activities of a large number (more than 1700) of histones and non-histone proteins, among them tubulin, p53, Hsp90, and NFYA (nuclear transcription factor Y subunit alpha) [16].

##### Mechanism(s) of acetyl transfer to lysine residues

**Scheme 2:**
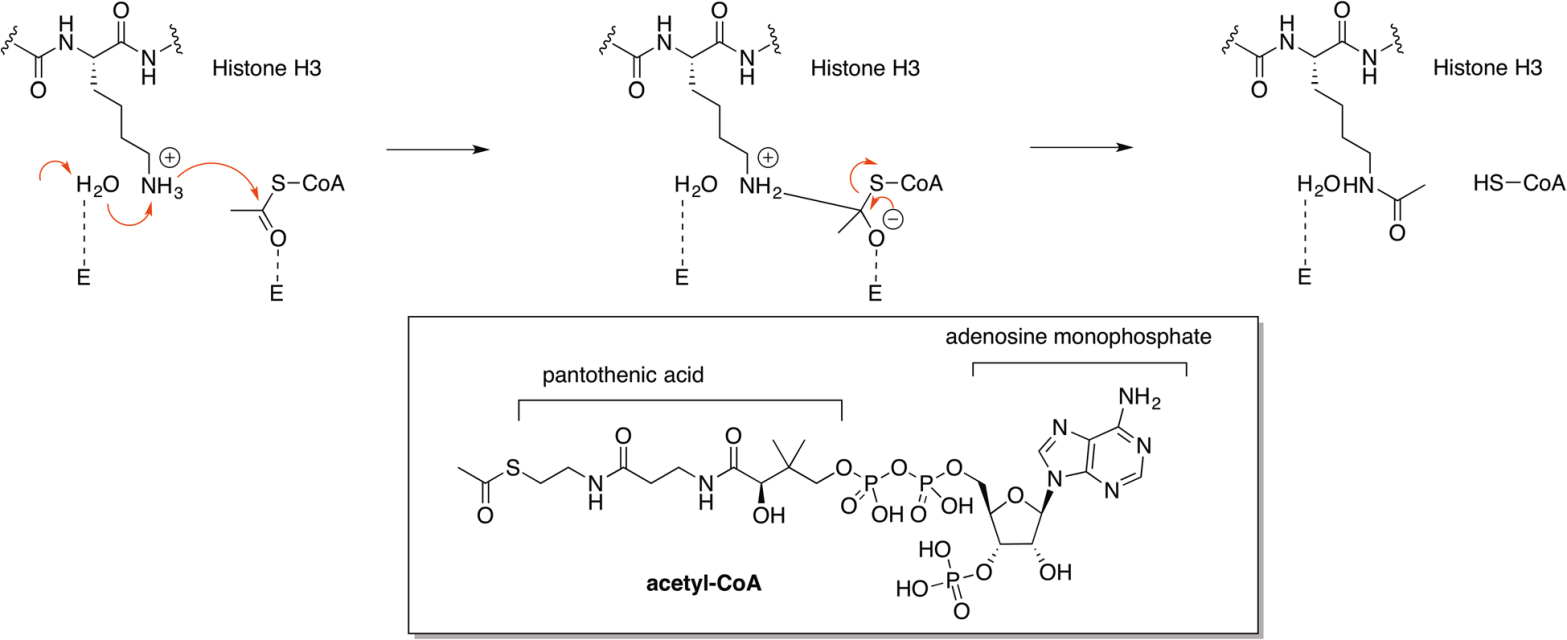
Mechanism of acetyl transfer in the ternary complex containing the HAT, acetyl-CoA (insert), and a fragment of H3 [17]

HATs catalyze the transfer of acetyl groups to lysine residues using acetyl-CoA as donor. Scheme 2 depicts the transfer of the acetyl group to the lysine **ε-**amino residues in histones on a ternary complex with the lysine substrate bound to a hydrophobic pocket located close to the acetyl group of the acetyl-CoA binding site, which is one of the mechanisms proposed based on crystal structures [17].

##### Mechanism(s) of acetyl-lysine hydrolysis by Zn^2+^-dependent deacetylases

The histone deacetylase family is composed of 18 members [18], which are divided into two groups depending on their mechanism of action: the classical Zn^2+^-dependent enzymes (HDAC1-11) and the NAD^+^ cofactor-dependent enzymes (SIRT1-7). The metalloprotein HDACs can be further classified into three groups: class I (HDAC1–3 and 8); class II (HDAC4–7 and 9–10), which may be divided into two subclasses, class IIa (HDACs 4, 5, 7, and 9) and class IIb (HDACs 6 and 10) on the basis of evolutionary relationships; and class IV, composed of HDAC11. Differences between class I and II HDACs are primarily noted in their size (with class II being from two to three times larger), their cellular localization, the conservation of sequence motifs in their catalytic domains, the identity of the protein-protein interaction complexes, and their tissue distribution.

**Scheme 3:**
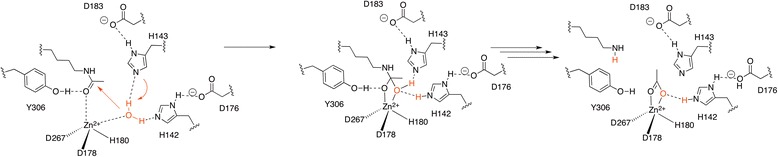
Simplified mechanism for HDAC-8 catalyzed deacetylation reactions [22]

Based on the ligand-bound crystal structures, the mechanism of deacetylation (Scheme 3) was recognized to involve the activation of the acetamide carbonyl group by the Zn^2+^ ion and its hydrolysis with formation of a tetrahedral intermediate facilitated by a “charge-relay” system. Several variants of the deacetylation mechanism have been proposed [19–22]. The most recent computations support the involvement of two charge-relay systems, the recognition of the H142/D176 dyad as the general base of the reaction, the stabilization of the intermediate by Y306, and the inhibitory effect of K^+^ (Scheme 3).

HDAC inhibitors [23] emulate the native acetylated lysine using a Zn^2+^-chelating “head group” attached via a connector of variable length and functionality to a cap region. The Zn^2+^-chelating “head groups” reported in HDACis includes virtually all functionalities known to bind transition metal ions (hydroxamic acids, thiols, mercaptoamides, trifluoromethylketones…), which most likely compete with the natural substrate after binding site occupancy [23]. For general inhibition by hydroxamic acids, a spontaneous proton transfer to an active site histidine upon binding of the inhibitor to the zinc was supported by recent computations; accordingly, for thiol-containing inhibitors (or precursors such as disufides or thioesters), the thiolate appears to be the active species [22].

##### Mechanism(s) of acetylated lysine deacetylation by sirtuins

**Scheme 4:**
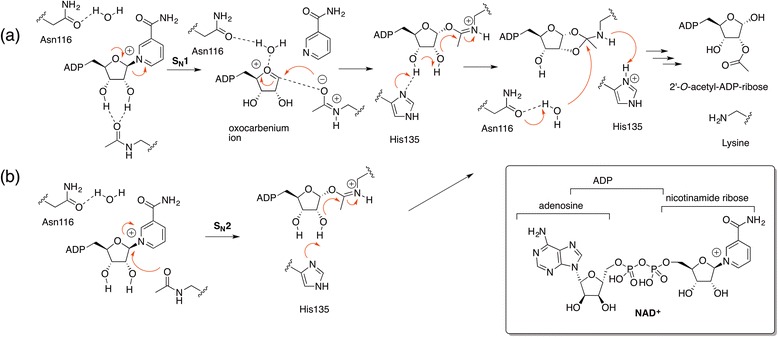
Mechanism of deacetylation of acetylated lysine catalyzed by sirtuins [24, 25, 30]. Insert is the structure of the cofactor NAD^+^

Catalytic mechanisms of nucleophilic substitution S_N_1-type [24, 25] or S_N_2-type [26–28] deacetylation by NAD^+^-dependent class III deacetylases or sirtuins [29] have been proposed with formation of an *O*-alkylamidate intermediate as shown in Scheme 4. A highly dissociative and concerted displacement of nicotinamide has been proposed as first step of the mechanism of deacetylation. The transition state shows a significant oxocarbenium ion character, but the cleavage appears to be facilitated by the nucleophilic assistance of the acetylated lysine, as shown by dynamics simulations [30].

#### Writers/erasers of methyl groups

##### Mechanism of methyl transfer catalyzed by DNMTs

**Scheme 5:**
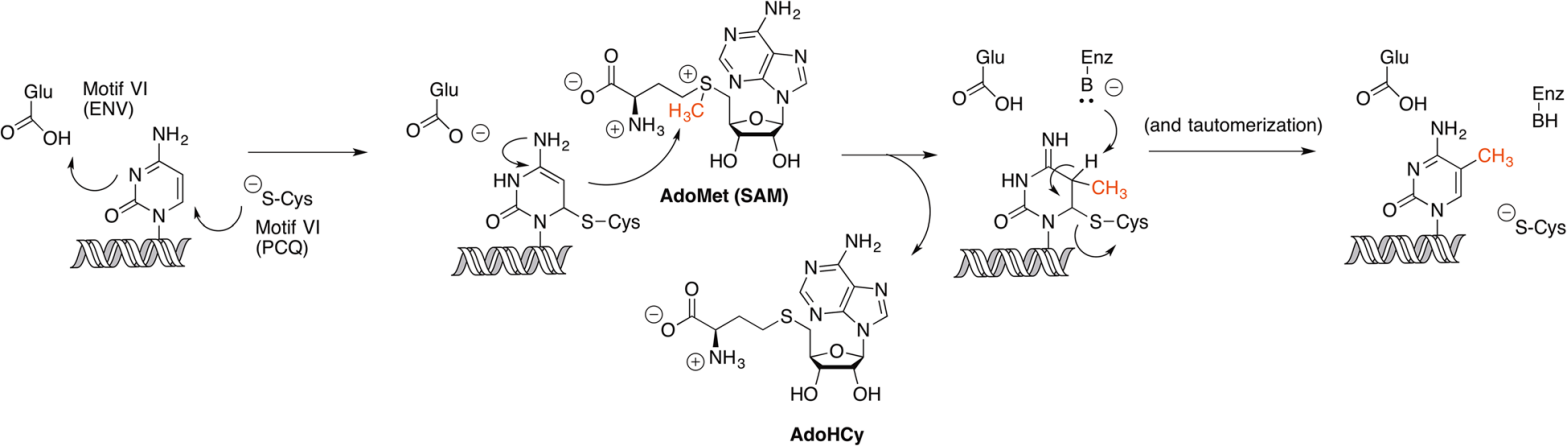
Mechanism of cytosine methylation at C5 catalyzed by DNMT, with SAM as electrophile

A mechanistic proposal for the DNA methylation at the cytosine C5 position in CpG nucleotide islands catalyzed by DNMT is shown in Scheme 5. The formation of a reactive enamine intermediate by the addition of a cysteine residue of the DNMT binding pocket to cytosine C6 position following base-flipping [31, 32], assisted by the protonation at C3 by a glutamic acid, is followed by the transfer of the methyl group of cofactor SAM to and a β-elimination on the 5-methyl-6-Cys-*S*-5,6-dihydrocytosine intermediate.

##### Mechanism of methyl transfer catalyzed by HMTs

**Scheme 6:**
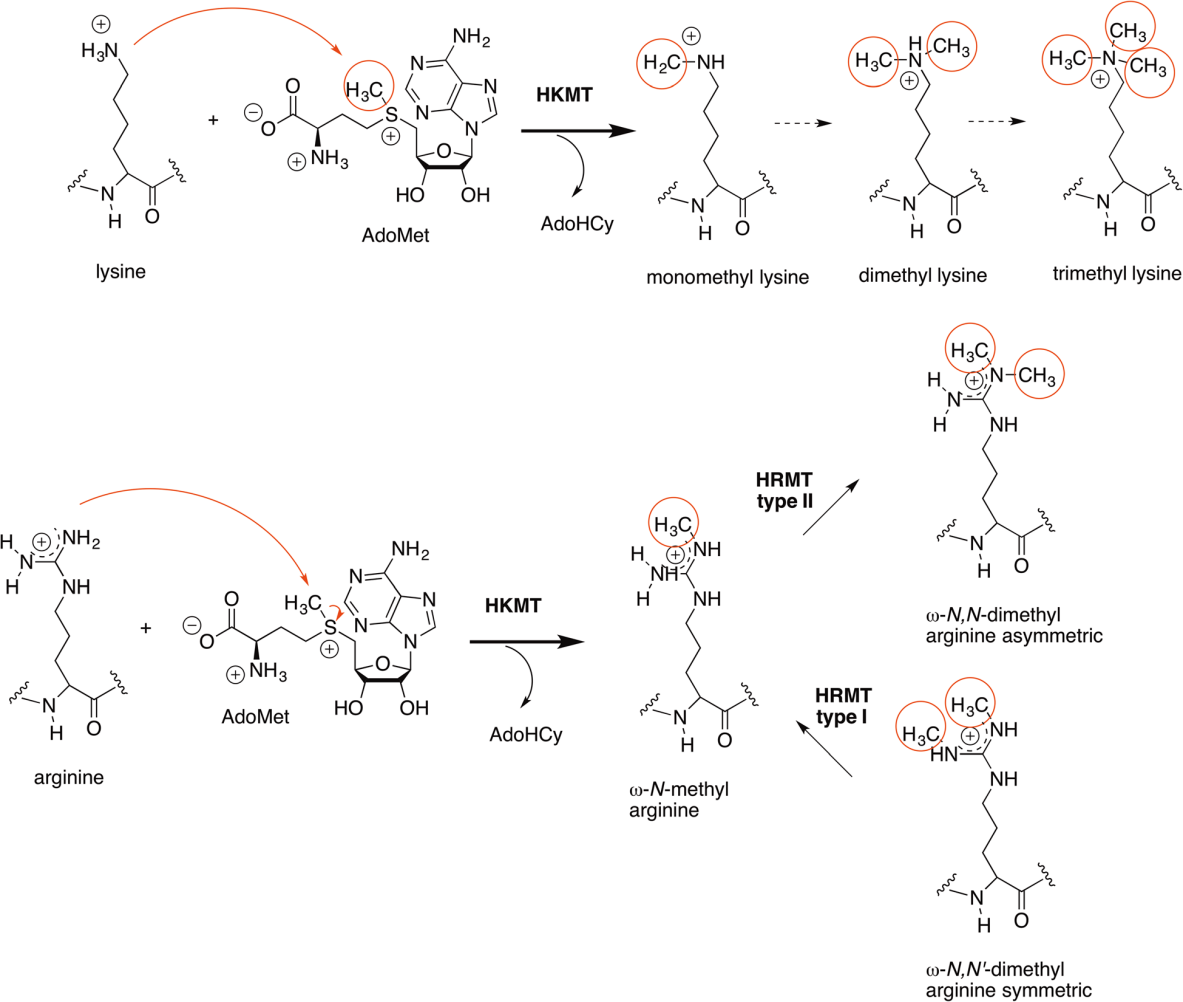
(*top*) Mechanism of methylation of histone lysine residues catalyzed by KMTs [35, 37] and (*bottom*) of arginine residues catalyzed by PRMTs [37]

The mechanism of methyltransferases of arginine and lysine residues of histones [33] is a classical nucleophilic substitution reaction of the methyl group donor SAM (Scheme 6) by the partially deprotonated terminal amino group of the basic amino acids, thus releasing *S*-adenosylhomocysteine (SAH) from the cofactor [34]. Computational studies of SET7/9, a monomethyltransferase (H3K4), revealed an in-line S_N_2 mechanism via a transition state of 70 % dissociative character [35]. More recent computations based on kinetic isotope effects are consistent with a S_N_2 mechanism involving the methyl transfer as the first irreversible step, with a transition state where the leaving group departure is retarded (2.5 Å) relative to the bond formation (2.1 Å) by the attacking nucleophile [36].

Similarly, the addition of methyl groups to arginine residues catalyzed by PRMTs uses SAM as cofactor but can produce mono- and/or dimethylarginine derivatives, the latter as the symmetric or non-symmetric isomers (Scheme 6) [37].

Nature uses two unrelated mechanisms for the removal of methyl groups from the methylated lysine and arginine residues [38, 39], each catalyzed by different demethylase enzymes [40]: (a) lysine specific demethylase 1 (LSD1/KDM1) and (b) Jumonji JmjC domain-containing demethylases (JHDMs).

**Scheme 7:**
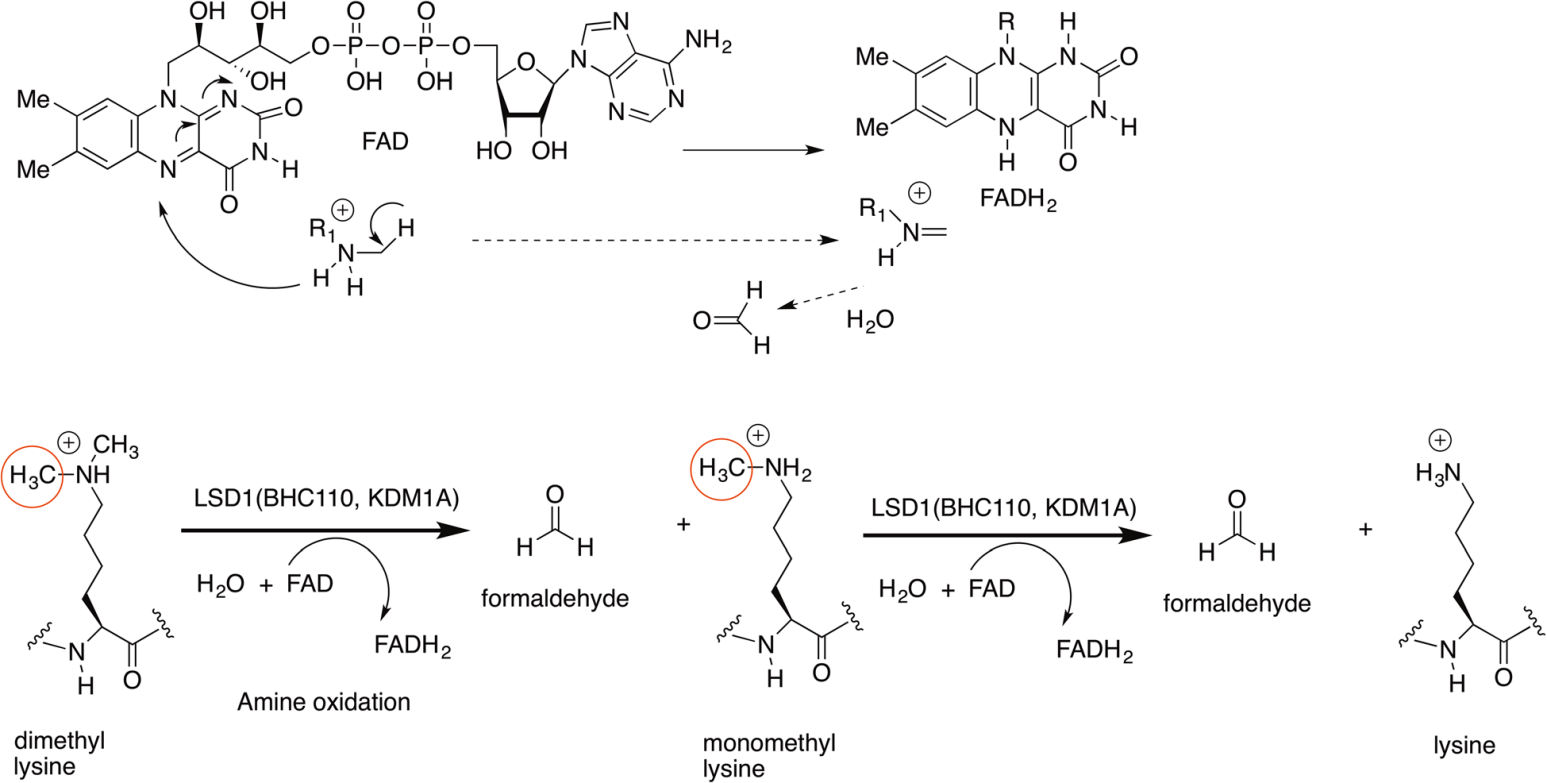
Mechanism of lysine demethylation catalyzed by LSD1 (BHC110, KDM1A)

The demethylation mechanism proposed for the LSD1/KDM1 demethylase starts with the oxidation of a protonated mono- or dimethylated lysine by oxidative cleavage of the α-CH bond of the substrate to form an iminium ion intermediate, with concomitant reduction of the cofactor flavin adenine dinucleotide (FAD) to FADH_2_, which is then reoxidized by molecular oxygen producing H_2_O_2_ (Scheme 7). The iminium ion intermediate is then enzymatically hydrolyzed to produce a carbinolamine, which releases formaldehyde and the demethylated lysine residue. The precise mechanism of imine formation is subject to debate, and either hydride or single electron transfer has been proposed for this step [41–43].

**Scheme 8:**
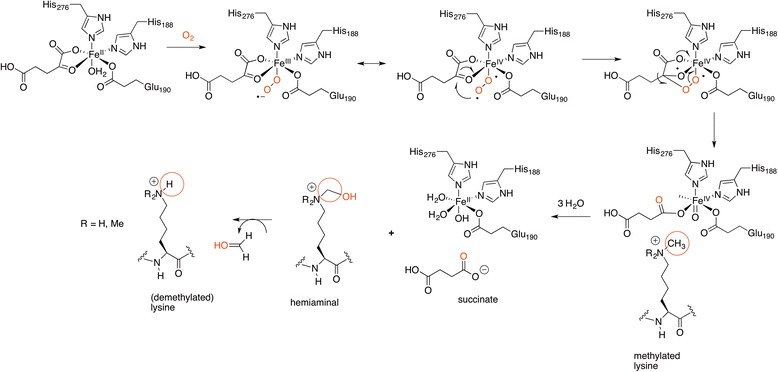
Mechanism of *N*-methyl lysine demethylation by the JHDM enzymes. The numbering is that of JMJD2A/KDM4a, which catalyzes demethylation of H3K9me2, H3K9me3, and H3K36me3

The oxidative demethylation reaction catalyzed by the JmjC family, similar to those catalyzed by dioxygenases that use Fe(II), takes place in a ternary complex containing succinate, the Fe(IV)-oxo complex, and the methylated lysine as shown in Scheme 8 [44–46]. First, the α-ketoglutarate complexed Fe(II) transfers an electron to the coordinated oxygen, giving rise to a peroxide anion (superoxide radical) and Fe(III). Nucleophilic attack of the anion to the carbonyl group (C2) of α-ketoglutarate produces an Fe(IV) bicyclic peroxyhemiketal and the intermediate undergoes decarboxylation to succinate. A highly unstable oxo-Fe(IV) intermediate is generated, and the oxoferryl group abstracts a hydrogen atom from the methyl group of *N*-methylated lysine, forming a Fe(III) hydroxide. Then, the radical recombination generates a carbinolamine that releases formaldehyde and the demethylated peptide.

#### Readers

The bromodomain and extra terminal domain (BET) family of tandem bromodomain-containing proteins (BRD2, BRD3, BRD4, and BRDT) exhibit a wide variety of biological effects and are the first readers to be targeted in epigenetic drug discovery. They are promising agents for the treatment of a spectrum of human diseases, ranging from cancer and inflammation to viral infections [47–49]. The binding of small molecules to the acetyl-lysine pocket (KAc) can block the recognition of their acetylated partner proteins via protein-protein interactions. Despite their overall structural similarity [47–49], subtle differences exist between the bromodomain structures and their functions that can account for their specificity.

### Epi-drugs in the clinic

The first drugs targeting epigenetics in fact predated a clear understanding of such mechanisms at the molecular level and the identification of the proteins responsible. Instead, the early compounds were advanced through the drug discovery process on the basis of their phenotypic effects in cancer models without knowledge of the precise targets.

The recognition that analogues of purine and pyrimidine nucleosides might act as anti-metabolites that dirsupt nucleic acid biosynthesis or function led to many such molecules being investigated. At the Czech Academy of Sciences, Piskala and Sorm synthesized 5-azacytidine (**18**) and the corresponding deoxyribose analogue **19** and demonstrated antileukemic activity in cells and AKR mice. Clinical trials with **18** began in 1967 in Europe and in 1971 an Investigational New Drug application was filed with the American National Cancer Institute. This was however rejected due to an unacceptably high level of toxicity. Interest in these nucleosides was rejuvenated due to the 1980 publication by Jones identifying DNMTs as their molecular target. Both **18** and **19** went back into clinical trials for the treatment of myelodysplastic syndrome, a hematological stem cell disorder that frequently progresses to acute myeloid leukemia, and were approved by the FDA in 2004 and 2006, respectively [50]. The nucleosides **18** and **19** are pro-drugs that enter the cell through transporters and are metabolically converted to the 5′-triphosphate of **19**. The triphosphate is incorporated into DNA strands, recognized as a cytosine substrate by DNMTs, and forms a covalent adduct with the enzyme via addition of the active site Cys residue to C-6 of the azapyrimidine heterocycle (see Scheme 5). The drugs are thus irreversible DNMT inhibitors, but their lack of selectivity between DNMT isoforms may be one reason for the high toxicity observed in settings other than myelodysplastic syndrome.

**Fig. 1:**
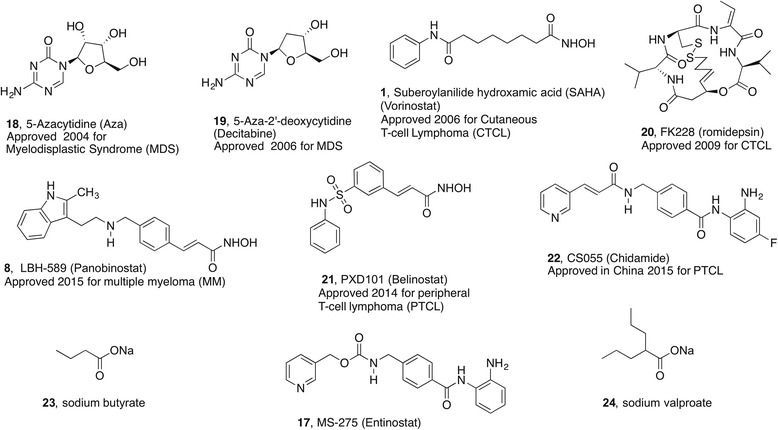
Epi-drugs approved for therapy (**1**, **8**, **18**–**22**), other drugs with epigenetic activities (**23**, **24**), and a candidate (**17**) undergoing advanced clinical studies

The first clinically approved inhibitors of zinc-dependent HDACs, vorinostat (SAHA, **1**) and romidepsin (**20**), were similarly discovered on the basis of their antiproliferative effects in cancer cells. Vorinostat evolved from DMSO as a lead for the differentiation of murine leukemia cells while romidepsin was identified in a screening campaign for compounds that reverse the phenotype of *ras*-transformed cells [51]. These compounds, like the other HDAC inhibitors displayed in Fig. 1, reversibly occupy the enzyme active site with the dominant interaction being coordination to the zinc cation (see Scheme 3) [52]. The most popular zinc-binding motif in synthetic HDAC inhibitors is a hydroxamic acid as in vorinostat and more recently approved agents panobinostat **8** and belinostat **21**. Another widely used zinc-binding group in medicinal chemistry efforts towards HDAC inhibitors is the benzamide as in chidamide **22** recently approved in China and entinostat **17** currently in clinical trials. Meanwhile, sodium butyrate **23** was in fact reported by several groups in 1977 and 1978 to increase the acetylation levels of histones through the inhibition of deacetylation. This led to the repurposing of sodium valproate **24**, an antiepileptic drug that primarily works through its action on voltage-gated sodium channels, as an HDAC inhibitor. At this point of time, such short chain carboxylic acids have yet to receive clinical approval as anticancer agents and their level of HDAC inhibition is modest compared to the hydroxamic acids and benzamides. Compared to the other clinical HDAC inhibitors, romidepsin **20** is unique in that it is a natural product rather than of synthetic origin. Furthermore, it is a disulfide prodrug that undergoes reduction in vivo to release a free thiol that acts as the zinc-binding group. Unlike vorinostat that is a pan-HDAC inhibitor, romidepsin is selective for class I isoforms.

### Combination therapies with epi-drugs towards polypharmacology

Current clinical practice uses drug combination therapies rather than single drugs [4] to treat patients with complex diseases [53]. The first clinical success with combination chemotherapy for childhood acute lymphoblastic leukemia (ALL) consisted of the co-administration of the anti-folate methothrexate, the tubulin-targeting vincristine (a *Vinca* alkaloid), the antimetabolite 6-mercaptopurine and the steroid prednisone. Either such a drug cocktail containing two or more individual tablets to combine therapeutic mechanisms or the co-formulation of two or more agents in a single tablet are the traditional modalities of drug combinations. The design of a drug combination aims to simultaneously block disease-related targets and is expected to ensure a more durable control of the disease progression compared to single agents. Therefore, the individual drugs should be active against their own target and ideally elicit synergistic effects when used in combination without increasing the toxicity and reducing drug resistance. Mathematical models have been recently developed that analyse the dynamics of pairs of drugs in a weighted linear superposition in order to obtain predictive drug effects (synergy, independence, antagonism…) from their use as multidrug and multidose combinations [54].

Following the trait mentioned above for the treatment of ALL, numerous combination therapies have been investigated for treating complex pathologies such as cancer, parasitic diseases, and multiple sclerosis that are polygenic in nature and result from the deregulation of complex protein networks. New drugs in the market, in particular those with a defined mechanism of action or target, are studied in combination even before they are launched. For example, in cancer, there are a large number of clinical studies that combine the proteasome inhibitor bortezomib with other drugs targeting not only the epigenome but also Hsp90, kinases, farnesyltransferases, etc., for both solid tumors and leukemias.

Post-genomic research over the last decade is shifting the focus of rational combination modalities to what is called “personalized medicine.” In the case of cancer, it involves targeting pathogenic oncogene and non-oncogene addictions, synthetic lethalities, and other vulnerabilities, attacking complementary cancer hallmarks or distinct cell populations with molecular targeted agents and using in addition other therapeutic options such as cytotoxic chemotherapy [55].

Despite the success of HDACis as single agents in the treatment of hematological maligancies, the treatment of patients with solid tumors has demonstrated limited clinical benefit [56]. For example, vorinostat **1** failed as monotherapy for the treatment of metastatic breast cancer in clinical trials [57]. This failure has prompted the investigation of novel treatment combinations with other cancer therapeutics, including kinase inhibitors, DNA-damaging chemotherapeutic agents, radiotherapy, hormonal therapies, and other epi-drugs (primarily DNA methyltransferase inhibitors), for which a rationale has been described [58].

In the case of tyrosine kinase inhibitors (TKIs), combination and multitarget therapies, including epigenetic drugs, are being developed since a large number of patients do not respond to single therapy or develop resistance. The results are encouraging. Vorinostat **1** and sorafenib **25** appear to interact in a synergistic fashion to kill carcinoma cells by activating CD95 through generation of ROS due to induction of cytosolic Ca^2+^ that elevates dihydroceramide levels [59]. Vorinostat **1** and other antagonists of receptor tyrosine kinase induced a synergistic induction of growth inhibition and apoptosis in the treatment of non-small cell lung cancer (NSCLC) (NCT00251589) (NCT00503971). The HDACi MPT0E028 **45** (shown in Fig. 4 below) enhances erlotinib (**26**)-induced cell death in epidermal growth factor receptor-tyrosine kinase inhibitors (EGFR-TKI)-resistant NSCLC cells [60]. Combination of EGFR-TKIs with vorinostat **1** resulted in significantly decreased cell viability through the activation of the apoptotic pathway and caspase-independent autophagic cell death [61].

Combination of vorinostat **1** with second-generation TKIs such as afatinib **27** or third-generation TKIs including WZ4002 **28** enhanced anti-tumor effect on xenografts of H1975 cells in vivo. The combination of new generation EGFR-TKIs and vorinostat **1** may be a new strategy to overcome the acquired resistance to EGFR-TKIs in T790M mutant lung cancer [61].

Synergistic effects of vorinostat **1** or sodium butyrate **23** with imatinib **29**, an ABL kinase inhibitor that can kill Breakpoint cluster region—Abelson (BCR-ABL) positive chronic myeloid leukemia (CML) cells, were observed and shown to enhance apoptosis in BCR-ABL expressing CML cells. The combination treatment was also effective against imatinib-refractory CML. Both wild-type BCR-ABL and the T315I mutant form of BCR-ABL, which is resistant to imatinib, were equivalently degraded following that combinatorial treatment [62, 63].

**Fig. 2:**
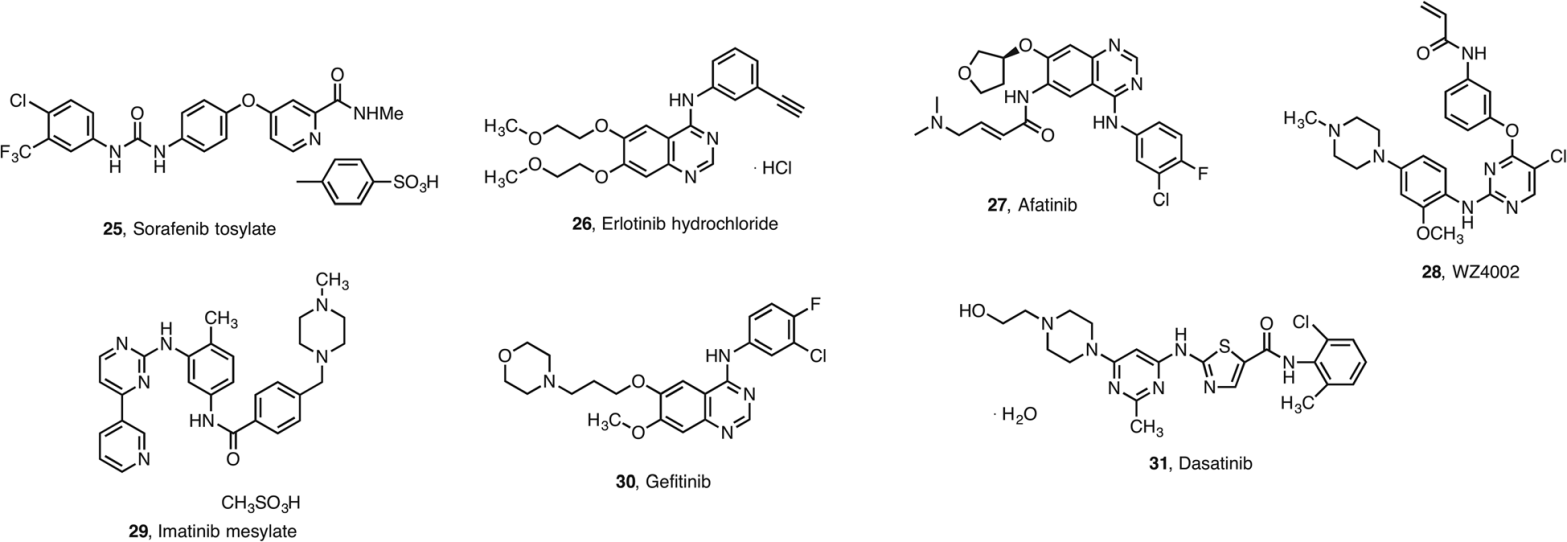
Selection of TKIs used in combination therapies with epi-drugs

Vascular endothelial growth factor (VEGF) and its receptor vascular endothelial growth factor receptor (VEGFR)-2 or kinase insert domain receptor (KDR) are key regulators of angiogenesis, which plays a key role in the growth of solid tumors and contributes to the progression of cancer metastasis. A phase I study of vorinostat **1** and VEGFR inhibitor gefitinib **30** (Fig. 2) in combination therapy has been approved for targeting resistance by B cell chronic lymphocytic leukemia-lymphoma-like 11 gene (BIM) polymorphysim in EGFR mutant lung cancer (VICTORY-J) (NCT02151721).

HDACis have been shown to downregulate estrogen receptor (ER) and androgen receptor (AR) mRNA in receptor-positive breast and prostate cancer cells [64–66]. Current drug therapies include tamoxifen **32** and raloxifene **33**, competitive ER inhibitors that act as selective estrogen receptor modulators (SERMs), and pure anti-estrogens such as fulvestrant **34**, which act as a selective ER downregulator (SERDs). Raloxifene **33** is an antagonist in all tissues, whereas tamoxifen **32** displays partial agonistic activity in a tissue and gene specific manner. In ER-negative cells, silenced ERs can be re-expressed using HDACi, restoring sensitivity to tamoxifen **32** [67]. HDACi increase the antitumor effects of tamoxifen **32** in several ER-positive breast cancer cell lines and breast tumors that are resistant to tamoxifen (NCT00365599) (NCT01194427) (NCT02395627). Co-treatment of breast cancer cells with HDACi and tamoxifen **18** produced a synergistic effect with depletion of both ER and progesterone receptor (PR), and this effect was exclusive of HDAC2-selective inhibitors [64]. In phase II clinical studies, the combination of vorinostat **1** and tamoxifen **32** is well tolerated by patients with ER-positive metastatic breast cancer progressing on endocrine therapy and exhibits promising activity in reversing hormone resistance. A 19 % objective response rate and a 40 % clinical benefit rate were noted [68].

**Fig. 3:**
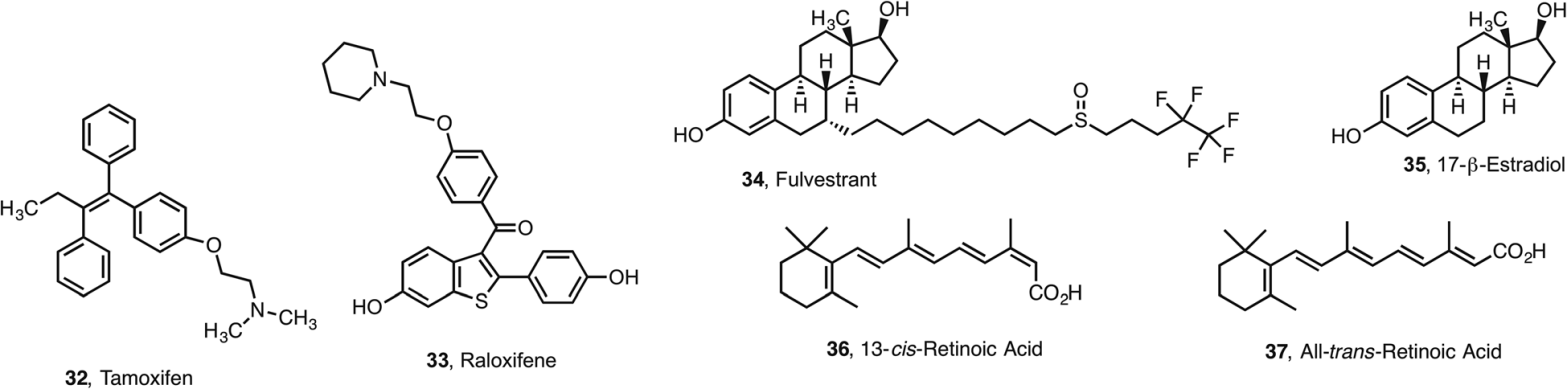
Selection of modulators of NRs used in combination therapies with epigenetic drugs

HDACi have shown antiestrogenic activity in human MCF7 breast cancer cells. The effect of the HDACis sodium butyrate **23** and vorinostat **1**, alone and in combination with 17β-estradiol (E2) **35** and the pure anti-estrogen fulvestrant **34** was examined. HDACis were found to antagonize the effect of E2 on the expression of cell cycle proteins, cell growth, and transcription of ER-dependent genes as a consequence of downregulation of the expression of ERα and prevention of receptor phosphorylation [69]. Thus, the combination of anti-estrogens with HDACi in clinical settings may improve efficacy while reducing side effects (Fig. 3).

A phase I study of the histone deacetylase inhibitor entinostat **17** in combination with 13-*cis*-retinoic acid **36** was carried out in patients with solid tumors, but no tumor responses were seen [70].

**Fig. 4:**
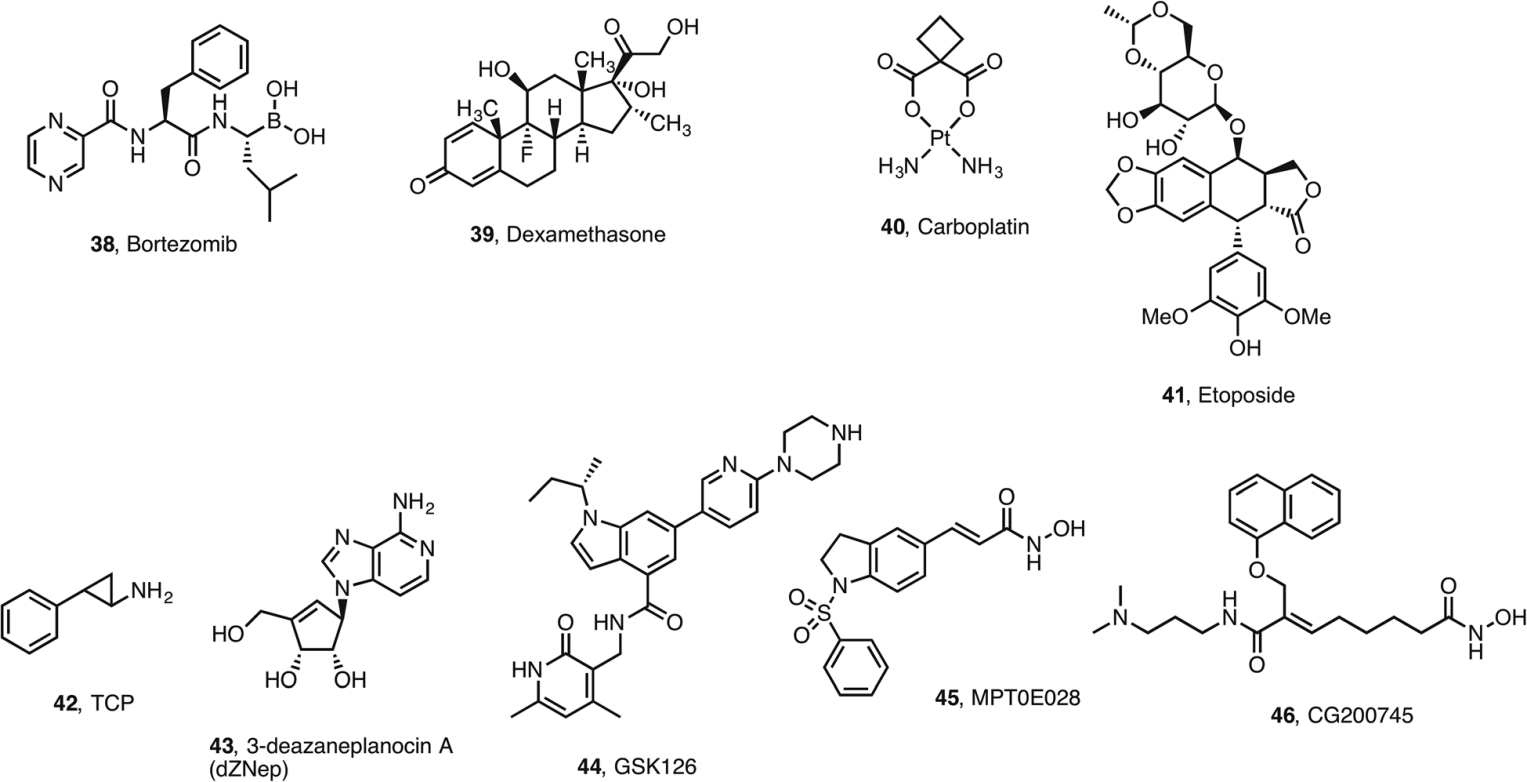
Selection of epi-drugs and other drugs used in combination

Vorinostat **1** in combination with the proteasome inhibitor bortezomib **38** (Fig. 4) resulted in synergistic antiproliferative and proapoptotic effects in colon cancer cell lines (NCT00574587) (NCT00258349) [71]. The same combination was found to block tumor cell growth in relapsed or refractory multiple myeloma (MM) patients (NCT00773747). The approval of panobinostat **8** for the treatment of MM patients was accelerated after the promising activity exhibited by its combination with bortezomib **38** and dexamethasone **39** (PANORAMA-1 phase III randomized clinical trial).

A phase I study has been initiated to evaluate the safety and efficacy of oral panobinostat **8** in combination with radiotherapy or chemoradiotherapy in patients with inoperable stage III NSCLC [72].

The approved DNMTi are likewise undergoing clinical studies in combination with other agents. Promising results have been obtained in the combination of DNMTi decitabine **19** plus TIK dasatinib **31** in phase I/II clinical studies in patients with CML (NCT1498445) [73]. Decitabine **19** combined with the DNA-damaging agents carboplatin **40** is in phase II clinical trials in platinum-resistant ovarian cancer (NCT00477386) [74].

The LSD1 inhibitor tranylcypromine (TCP, **41**) combined with all-*trans*-retinoic acid **37** (Fig. 3) is now in clinical trials for the treatment of adult patients with acute myeloid leukemia (AML) and myelodisplastic syndrome (MDS) (NCT02273102) and patients with relapsed or refractory AML (NCT02261779) in non-acute promyelocytic leukemia (APL) AMLs when treatment with all-*trans*-retinoic acid **37** is not effective.

The topoisomerase IIa (TopIIa) inhibitor etoposide **42** combined with the Enhancer of Zeste Homologous 2 (EZH2) inhibitors 7-deazaneplanocin A (DZNep) **43** or GSK126 **44** induces cell death in murine and human prostate cancer cell lines and showed therapeutic efficacy in vivo. Thus, the combination of a low dose TopIIa inhibitor with a EZH2 inhibitor is beneficial against aggressive prostate cancer [75]. Likewise, EZH2 inhibition sensitizes transcription activator BRG1 (ATP-dependent helicase SMARCA4) and EGFR mutant lung tumors to TopoII inhibitors, which suggest that combination therapy is a promising approach to this cancer [76].

Novel epigenetic modulators continue to reach clinical trials. For example, the first-in-man study of the toxicity, pharmacokinetics, and pharmacodynamics of CG200745 **46**, a pan-HDAC inhibitor, in patients with refractory solid malignancies was initiated in 2015 [77]. CG200745 **46** can be safely administered at effective dose levels that inhibit HDAC in peripheral blood mononuclear cells (PBMCs) and tumor tissue, although maximum tolerated dose (MTD) was not reached [77].

### Combinations of epigenetic drugs

In the investigation of novel treatment options, the simultaneous targeting of multiple epigenetic systems, notably when HDACi and DNMTi are administered together, aims to achieve efficient epigenetic gene reactivation (http://clinicaltrials.gov/). For example, results of the phase I/II trial of combined epigenetic therapy with DNMTi azacitidine **18** and HDACi entinostat **17** in extensively pretreated patients with recurrent metastatic NSCL are encouraging [78]. The combination of vorinostat **1** and cladribine (2-chlorodeoxyadenosine) **47** synergistically induced apoptosis in natural killer cell large granular lymphocytes (NK-LGL) leukemia [79]. Cladribine **47** is a drug approved for the treatment of hairy-cell leukemia and acts as indirect DNMTi, since it inhibits SAH hydrolase, increasing competition of SAH for the SAM binding site.

The combination of HDACi (and also other chromatin remodeling enzyme inhibitors such as DNMTi) with the lysine methyltransferase inhibitor DZNep **43** revealed the importance of pharmacological combinatorial approaches in breast cancer cells and in the regulation of cancer immunity [80]. Also encouraging are the results of triple combination using HDACi (TSA **48**), DNMTi (5-AZA-CdR, **19**), and EZH2 inhibitor (DZNep, **43**) on human AML cells [81]. The triple combination (which proved to be more effective than the combination of two agents or a single agent) induced a remarkable synergistic antineoplastic effect as demonstrated by an in vitro colony assay and also showed a potent synergistic activation of several key tumor suppressor geners (TSGs) as determined by real-time PCR.

The combination of vorinostat **1** and the LSD1 inhibitor tranylcypromine **40** was able to reduce glioblastoma stem cell viability and displayed efficacy in a U87 xenograft model [82].

BET inhibitors are also promising therapeutic agents [47, 49, 83], although resistance has been documented [84, 85]. Their efficacy might be explained by the chromosomal translocations involving bromodomains BRD3 and BRD4 occurring in NUT midline carcinoma (NMC) and in AML [86, 87]. BET inhibition led to promising results in mouse models of sepsis [88], autoimmunity (in combination with a Myc inhibitor) [89], and inflammation of the lung [90]. As an example, JQ-1 **49** [91] prevented tumor progression by promoting differentiation in murine NMC [91] and also cardiac hypertrophy in mice [92].

The combined inhibition of BET family proteins and HDAC has been considered as a potential epigenetics-based therapy for the treatment of pancreatic ductal adenocarcinoma [93]. Clinical trials have also been initiated for the treatment of relapsed or refractory neuroblastoma (NCT02337309) and dose escalation studies for intravenous infusions in patients with other solid cancers are underway (NCT00907205).

A combination of BET inhibitors (JQ-1 **49**) and SIRT activators (SRT1720, **51**) was found to alleviate inflammatory response due to the upregulation of SIRT1 by the BETi JQ-1 **49**, thus reversing the pro-inflammatory response to SIRT1 inhibition in a cellular lung disease model [94]. On the other hand, the combination of JQ-1 **49** with gamma-secretase inhibitors was shown to be effective against primary human leukemias in vivo [95].

**Fig. 5:**
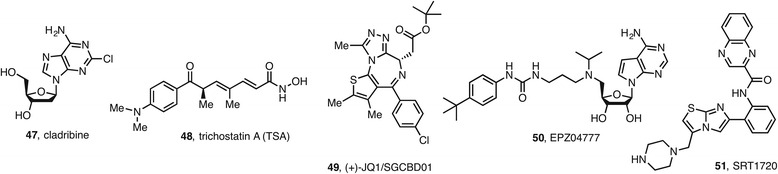
Selection of epigenetic drugs used in combination therapies

The inhibition of SIRT1-mediated epigenetic silencing of *MLL*-rearranged leukemia by disruptor of telomeric silencing 1-like (DOT1L) inhibitors confirmed that the combination of epigenetic drugs (DOT1L inhibitor EPZ04777, **50** and SIRT1 activator SRT1720, **51**) targeting the activation and repression of gene expression is also a promising approach to treat leukemia [96] (Fig. 5).

### Dual acting hybrids with an epigenetic and a second mechanism of action

Epigenetic therapies are coming of age, and seven drugs have been approved for cancer, with many more undergoing clinical trials. Advances in genome-wide analyses and bioinformatics are providing information on the disease-supportive and disease-irrelevant gene networks that are deregulated by aberrant epigenetic modifications. Features important for epigenetic therapies are well recognized: (a) epigenetic deregulation causes both gene specific and systemic effects; (b) crosstalk and complex formation occur between epigenetic modifiers, which implies that multiple epigenetic systems are likely to be affected [97, 98]; (c) genetic instability of cancer cells has the most likely altered multiple epigenetic systems at the time a patient is diagnosed; (d) the observations that existing epigenetic drugs affect normal cells less than cancer cells indicated either a higher epigenetic plasticity of normal cells or a particular sensitivity of tumor cells to certain epigenetic drug activities; (e) epigenetic drugs are in principle non-genotoxic and their action can be made reversible upon discontinuation of the treatment; (f) as for all drugs, the development of resistance to a single agent is a concern; (g) certain epigenetic drugs can “override” not only their cognate target but also the silencing caused by other epigenetic enzymes [99].

Together, the above aspects provide a rationale for the combination of pharmacophores, one of them targeting the epigenetic enzymatic machinery, and also for the simultaneous targeting of multiple epigenetic systems. Most of the epigenetic drugs developed following the first strategy are hybrid molecules containing the scaffold of an HDACi fused/linked to another anticancer drug, cytotoxic agent, anti-angiogenesis drug, etc., acting at a related target. There are two principal reasons for the popularity of HDACs in the design of dual targeting agents. Firstly, HDACs are the epigenetic targets that have received the most attention for drug discovery, and consequently, there are a multitude of high affinity inhibitors known with diverse chemical scaffolds. Secondly, the HDAC pharmacophore, backed up by X-ray cocrystal structures of enzyme-inhibitor complexes, includes a “cap” region that is protruding from the active site channel and engaged in binding interactions with the enzyme surface. While these are important, they are less dominant in driving potency compared to the coordination to the active site zinc cation. As a result, the surface-binding cap is tolerant of a high degree of structural variation without compromising HDAC binding. It is hence possible to incorporate a cap that contains the pharmacophore for a second non-HDAC target and the resulting chimeric molecule is capable of binding to both these targets.

**Fig. 6:**
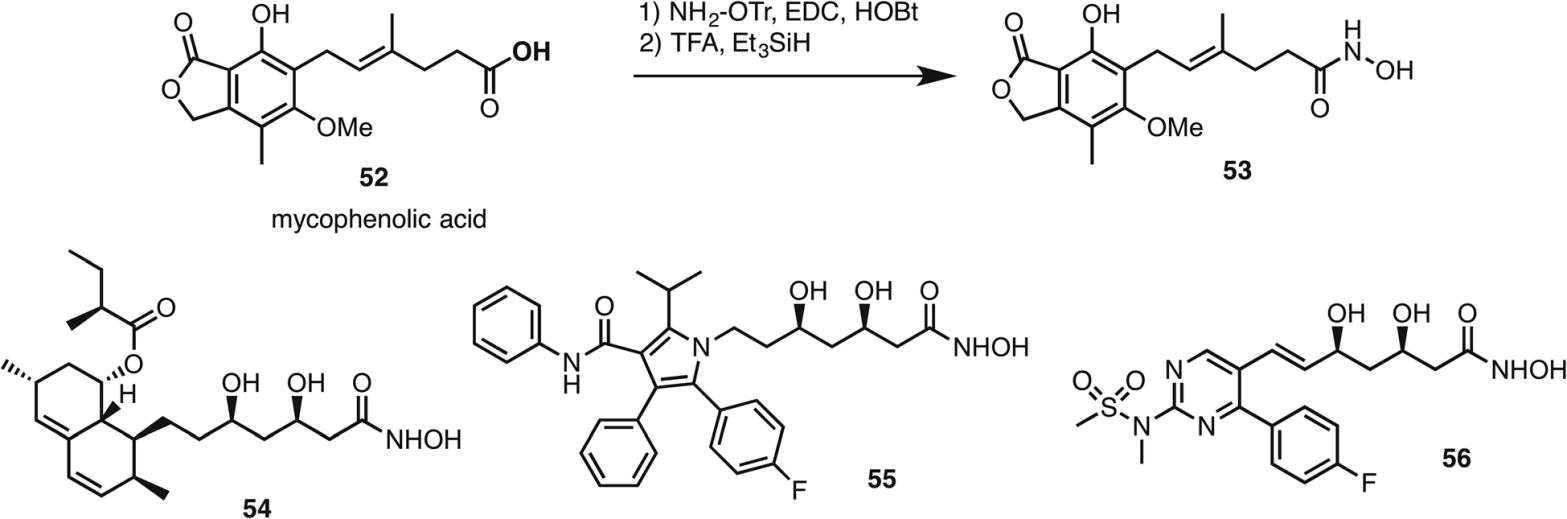
Examples of multitarget HDAC inhibitors obtained from drug molecules containing carboxylic acids

An early publication illustrating the multitarget principle was reported by Pankiewic in 2007. Mycophenolic acid **52** (Fig. 6) is an inosine monophosphate dehydrogenase (IMPDH) inhibitor clinically used as an immunosuppressant that contains a carboxylic acid functional group. By conversion to a hydroxamic acid, the analogue **53** was demonstrated to retain nanomolar activity against IMPDH while additionally acting as a micromolar HDAC inhibitor [100]. The analogue was slightly more active (IC_50_ 4.8 μM) than mycophenolic acid in the growth inhibition of K562 cell lines. In the same way, other drugs containing carboxylic acids or their equivalents could be converted to hydroxamic acids with the potential gain of HDAC inhibitory activity. Besides mycophenolic acid, another example involves the blokcbuster drug lovastatin, a 3-hydroxy-3-methylglutaryl coenzyme A reductase (HMGCR) inhibitor used as a colesterol-lowering agent. The corresponding lovastatin hydroxamic acid **54** was a nanomolar inhibitor of both HMGCR and HDACs and displayed efficacy in a colitis-associated colorectal cancer mouse model [101]. The authors additionally prepared the hydroxamic acid versions **55** and **56** of second-generation statins atorvastatin and rosuvastatin, respectively. Both compounds were nanomolar inhibitors of HDAC1, HDAC6, and HMGCR. In cell-based assays, there was evidence of dual target engagement in increased levels of acetylated histones and tubulin and decreased enzymatic activity of HMGCR. Despite the synthetic ease of taking known drugs containing carboxylic acids and converting them to hydroxamic acids, this approach has rarely been employed as a means to obtain HDACi gain of function in the resulting hybrid.

The most popular strategy for a dual action HDAC inhibitor consists of taking a known pharmacophore for a second target and grafting a side-chain containing a spacer and a zinc-binding group. This has been extensively studied with heterocyclic scaffolds that are protein kinase inhibitors. Part of the rationale comes from the synergy observed with kinase and HDAC inhibition in vitro and in vivo models that has spurred clinical trials featuring combination therapy as discussed above (“Combinations of epigenetic drugs”). Furthermore, just like HDAC inhibitors, kinase inhibitors often contain regions that are involved in improving pharmacokinetics rather than bonding interactions with the enzyme active site and are amenable to modification. Since resistance is a major issue with kinase inhibitors in vivo, the addition of an independent mechanism of action may help circumvent this problem.

**Fig. 7:**
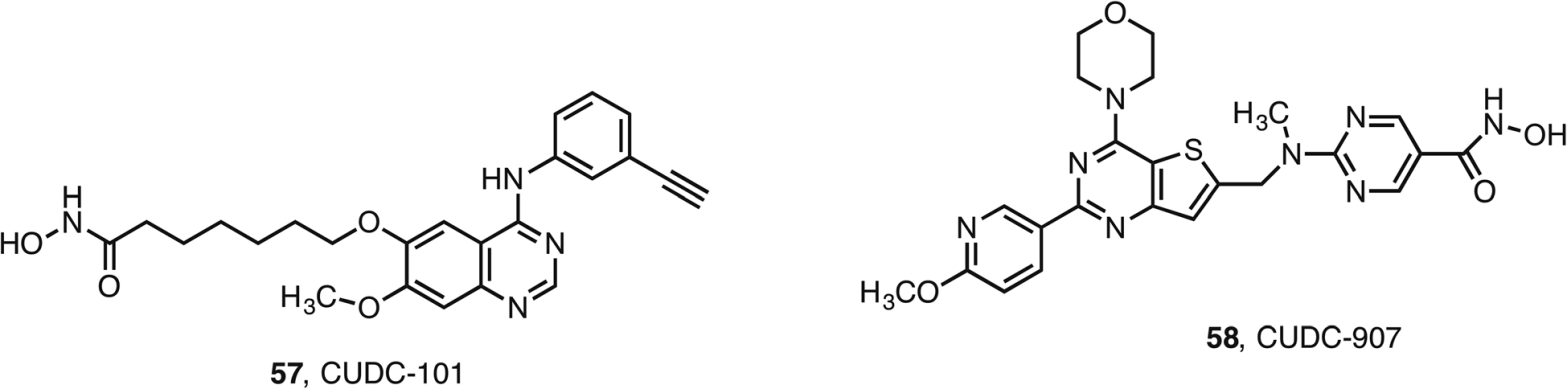
Dual HDAC and kinase inhibitors currently in clinical trials

A number of approved kinase inhibitors have served as an inspiration for the design of dual HDAC targeting agents viz. erlotinib **26** [102, 103], imatinib **29** [104], lapatinib [105], and vandetanib [106] as well as the clinical candidate semaxanib [107]. The most advanced of these hybrids, CUDC-101 **57** (Fig. 7), from Curis, recently completed phase I clinical trials in several forms of cancer [108]. The Curis approach was based on the X-ray cocrystal structure of erlotinib with EGFR that indicates key hydrogen bond interactions between N1 and N3 of the quinazoline heterocycle and the ATP binding domain of the kinase. Meanwhile, the solvent exposed phenoxy substitutents are protruding out of the active site and not involved in significant enzyme binding. The Curis scientists predicted that these positions should tolerate modification without loss of affinity and designed a series of compounds containing a zinc-binding hydroxamic acid and various spacers [109]. From this series, CUDC-101 emerged as the clinical candidate. It is a nanomolar inhibitor of the intended kinases (IC_50_ 2 nM for EGFR, 16 nM for HER2) while relatively inactive against other kinases tested. In addition, it is a nanomolar inhibitor of class I (IC_50_ HDAC1 4.5 nM, HDAC2 12.6 nM, HDAC3 9.1 nM, HDAC8 79.8 nM) and class II HDACs (IC_50_ HDAC4 13.2 nM, HDAC5 11.4 nM, HDAC6 5.1 nM, HDAC7 373 nM, HDAC9 67.2 nM) as well as HDAC10 (IC_50_ 26.1 nM). The promising data from phase I trials suggests that CUDC-101 will progress to phase II. In a separate program, Curis have applied the dual targeting philosophy to the non-protein kinase, phosphatidylinositol 3-kinase (PI3K). In this case, the pan-PI3K inhibitor pictilisib was the starting point and led to the hybrid CUDC-907 **58**. The compound is a nanomolar inhibitor of class I, II, and IV HDACs as well as all four PI3K isoforms [110]. CUDC-907 is currently in phase II trials and has received orphan drug for relapsed or refractory diffuse B cell lymphoma although there may be concerns about toxicity as observed with other pan-PI3K inhibitors.

**Fig. 8:**
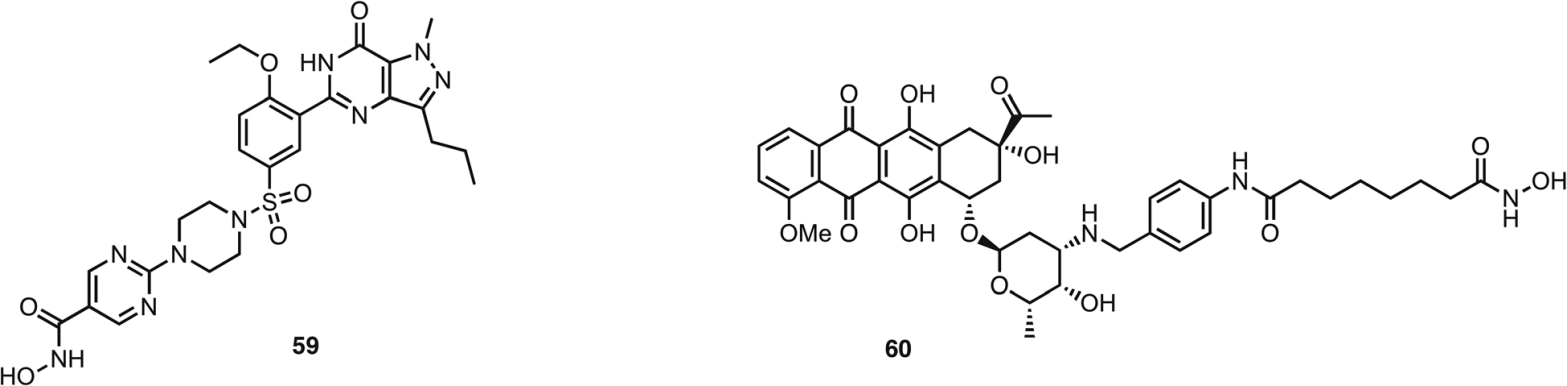
PDE and topoisomerase inhibitors with dual HDAC inhibitory activity

The inhibition of enzymes that are not protein kinases has also been succesfully combined with HDAC inhibition. A patent [111] describes the preparation of hybrid molecules based on the phosphodiesterase 5 (PDE5) inhibitor sildenafil (Viagra). The piperazine fragment in sildenafil occupies a hydrophobic pocket in the enzyme active site and can be altered without significant loss of binding. Attachment of a hydroxamic acid led to dual HDAC/PDE5 inhibitors exemplified by **59** (Fig. 8) that inhibits HDACs and PDE5 with an IC_50_ below 10 nM. In support of their application in Alzheimer’s disease, these sildenafil hybrids increase acetylated tubulin levels and decrease amyloid-β precursor protein and Tau phosphorylation, and cross the blood-brain barrier in a mouse model. Another family of enzymes that has been targeted are the DNA topoisomerases. A number of natural products including daunorubicin, camptothecin, and podophyllotoxin are topoisomerase inhibitors that are approved in their own right or led to semi-synthetic derivatives in clinical use. These natural scaffolds have been modified to attach a zinc-binding group, leading to dual HDAC inhibition in preclinical examples such as **60** [112]. This compound was prepared in one step from daunorubicin by reductive alkylation of the amine and inhibited the DU-145 cell line with an IC_50_ of 1.6 μM. In cell-based assays, HDAC inhibition was evidenced by increased levels of p21 and acetylated H4 and tubulin, while topoisomerase II inhibition was demonstrated in a DNA plasmid relaxation assay and formation of the trapped topoisomerase II-DNA cleavage complex at micomolar drug concentrations.

**Fig. 9:**
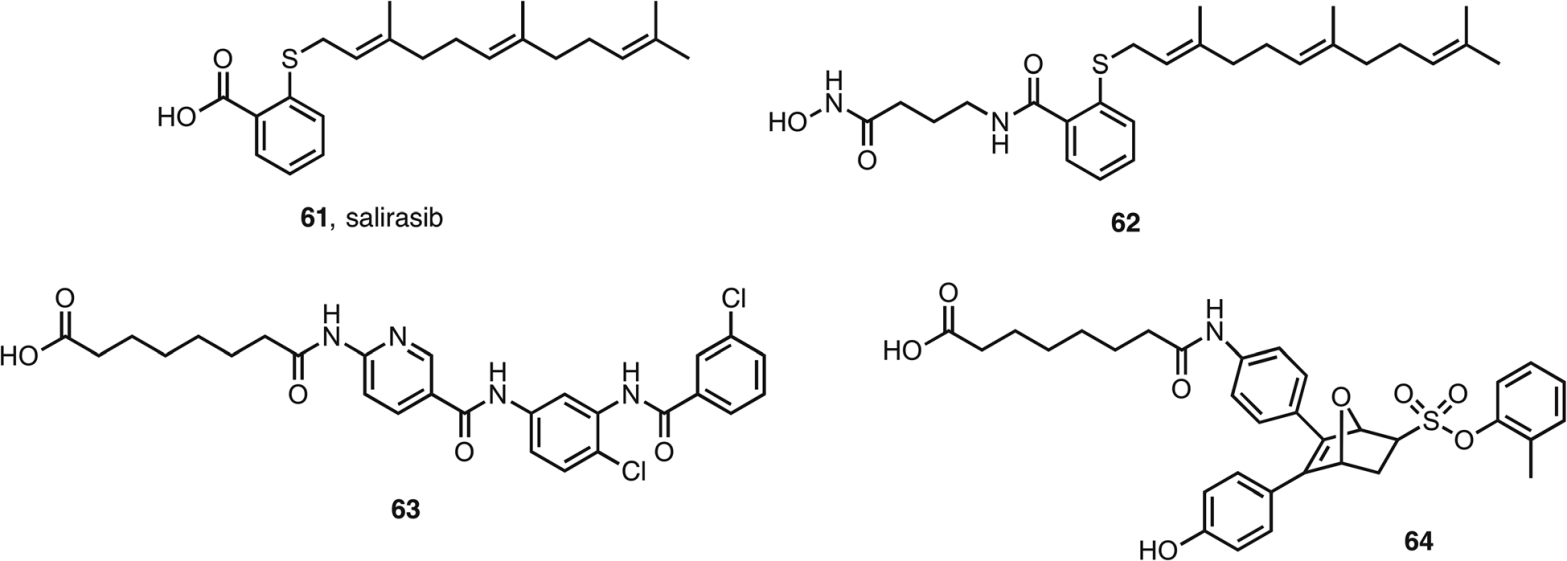
Protein receptor ligands with dual HDAC inhibition

Besides direct inhibition of enzymes, a different approach is the prevention of cellular localization to the appropriate compartment. For example, the Ras GTPase protein’s location in the cell membrane is inhibited by the drug salirasib **61** (Fig. 9). The hydroxamic acid containing conjugate **62** was a submicromolar inhibitor of HDAC1, HDAC6, and HDAC8 [113]. In cells, the compound increased acetylation levels of histones and tubulin and decreased signaling through the phospho-protein kinase B (pAkt) and phospho-protein kinase RNA-like endoplasmatic reticulum kinase (pERK) pathways.

The above examples illustrate the dual action against HDACs and a non-epigenetic enzyme. In the same way, it is possible to design HDAC inhibitors that are ligands for a non-epigenetic receptor. For example, the membrane protein smoothened is part of the Wnt/β-catenin signaling pathway and is targeted by the recently approved antagonist vismodegib. The hybrid molecule **63** is nanomolar in binding to the protein and inhibiting HDAC1, 2, 3 and 6 [114]. In cell-based assays, levels of acetylated histones and tubulin was increased whereas Gli-2 and Hedgehog signaling was decreased, supporting dual target engagement. A number of ligands for the nuclear hormone superfamily have been successfully modified to be dual HDAC inhibitory agents. For example, **64** is a submicromolar inhibitor of HDAC1 and the estrogen receptor and inhibited the MCF7 cell line with an IC50 of 5 μM [115]. The level of activity against HDACs is rather surprising as the compound contains a carboxylic acid rather than the usual hydroxamic acid as the zinc-binding group. Other groups have reported ligands for the vitamin D [116] retinoid X [117] and androgen [118] receptor that also inhibit HDACs.

**Fig. 10:**
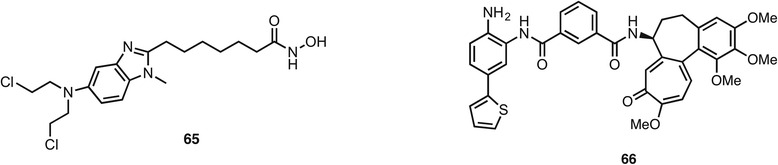
Examples of DNA targeting HDAC inhibitors

Outside the field of enzymes and receptors, the covalent alkylation of DNA has been combined with HDAC inhibition. Compound **65** (Fig. 10) is an analogue of the nitrogen mustard bendamustine that not only causes DNA damage in cells but also inhibits HDAC1 and 6 at nanomolar levels and showed efficacy in a HL60 xenograft model at a dose of 20 mg/kg [119]. Meanwhile, the natural product colchicine exerts an anticancer effect through disruption of tubulin polymerization. The colchicine analogue **66** inhibited HDAC1 and tubulin polymerization at micromolar levels and growth of the HCT116 cell line at a submicromolar level [120].

### Multitarget epigenetic modulators

**Fig. 11:**
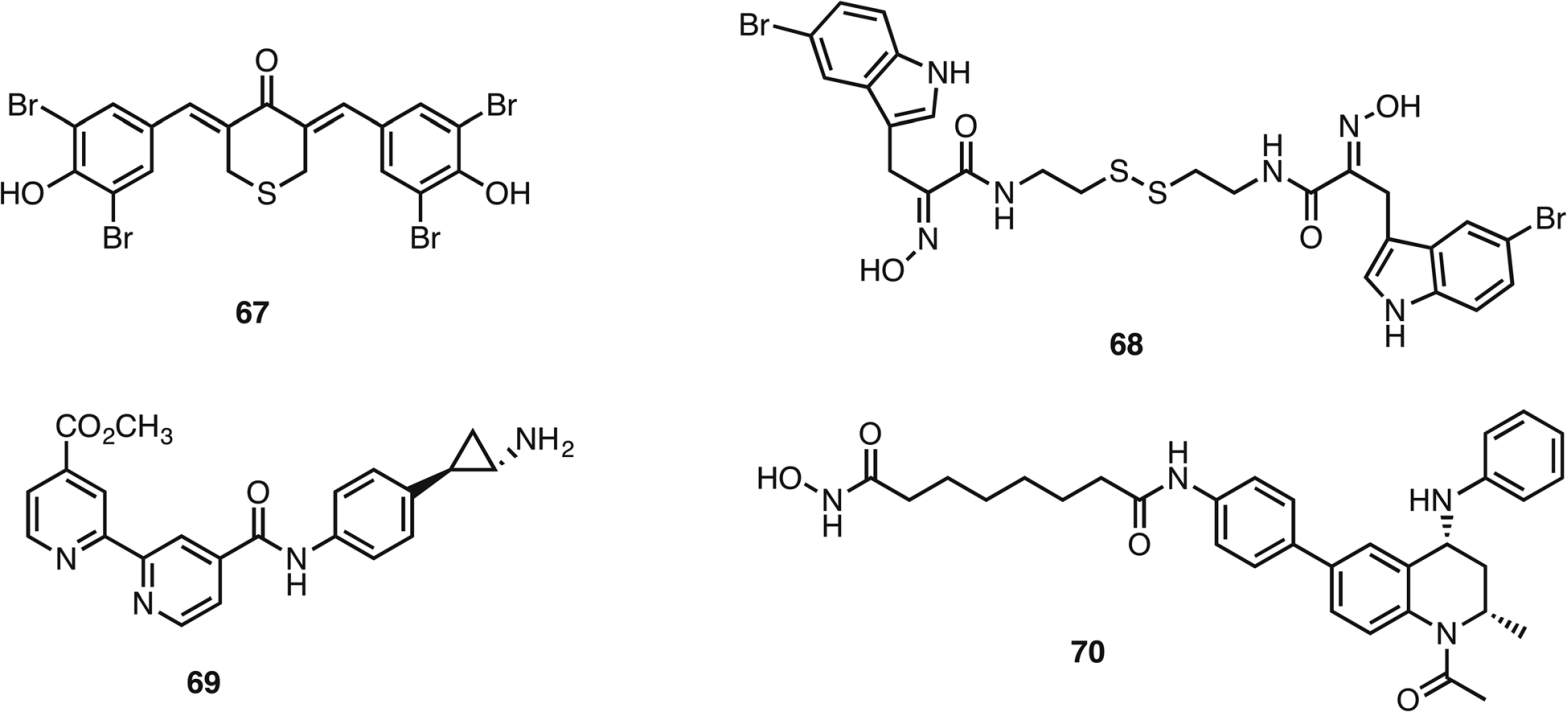
Examples of dual epigenetic targeting compounds

While the above examples have all involved one epigenetic and one non-epigenetic mechanism of action, it is possible to combine pharmacophores for multiple epigenetic targets in a single molecule. Two examples are compounds **67** (Fig. 11) and **68** that were inspired by the natural product scaffolds of curcumin and psammaplins respectively. Compound **67** affected histone methylation, acetylation and deacetylation [121] while **68** inhibited HDAC1, DNMT, and SIRT1 at the tested concentration of 1 μM [122]. Meanwhile, elaboration of the tranylcypromine **42** skeleton for lysin-specific demethylase inhibition to the analogue **69** with a metal binding motif accomplished additional inhibition of JmjC lysine demethylases, thus effectively acting as an inhibitor of lysine demethylation by both mechanisms of action [123]. At GlaxoSmithKline, a lead series for bromodomain binding was modified to enable dual HDAC inhibition. Compound **70** inhibited HDAC1 with an IC_50_ of 250 nM and bound to BRD4 with a K_d_ of 50 nM and increased H4 acetylation levels and decreased c-myc levels in cells [124]. However, the compound did not display synergy in its action over the combination of single agent HDAC and BRD inhibitors.

**Fig. 12:**
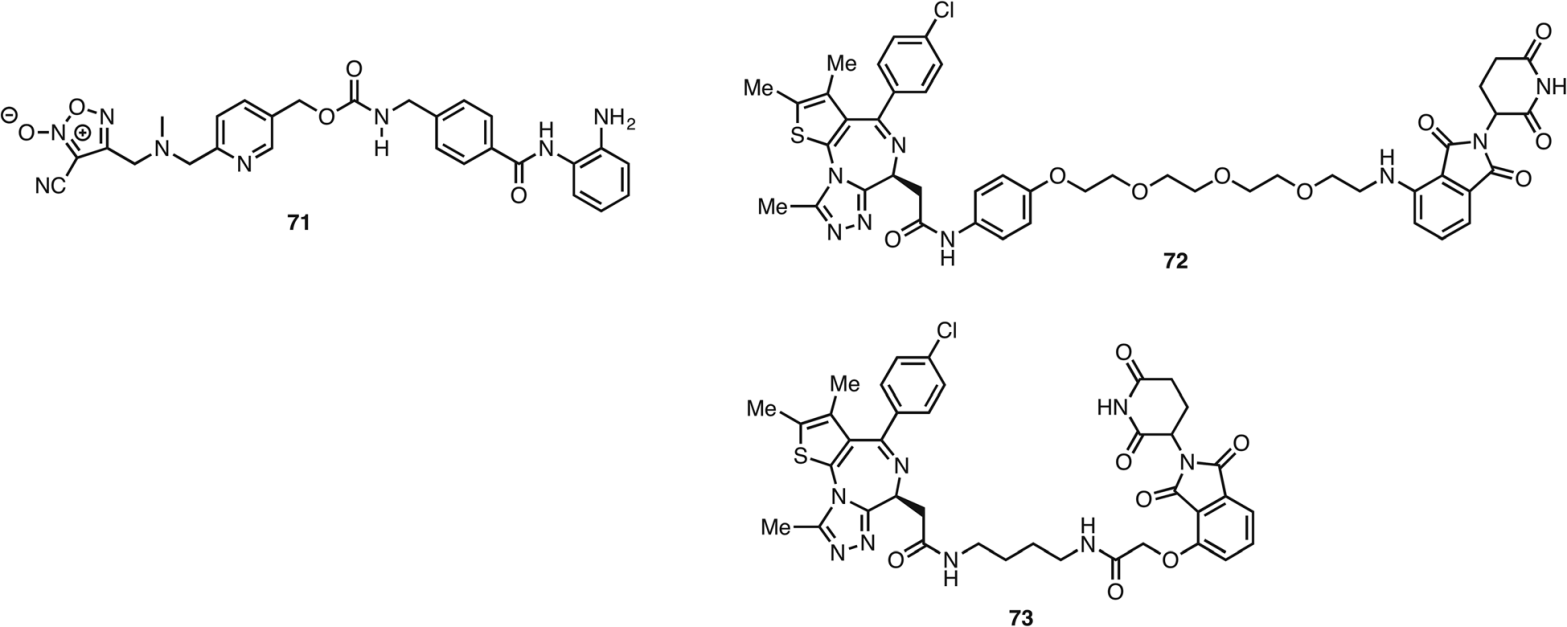
Compounds with a dual function to enhance an epigenetic mechanism of action

Finally, the purpose of dual targeting can be to enhance the effect upon the primary epigenetic mechanism of action. The clinical candidate HDAC inhibitor entinostat **17** was conjugated to a NO donor to give **71** (Fig. 12). In this hybrid, inhibition of HDACs was observed as well as an effect on cyclic GMP signaling and an increase in the post-translational *S*-nitrosylation of HDAC2 presumably due to the increased NO levels [125]. As discussed above, bromodomain ligands have attracted much attention as potential therapeutic agents. One issue, however, is that their effects can be transient due to compensation by increased expression of the targeted bromodomain. To overcome this problem, two groups have recently conjugated JQ-1 **49**, a bromodomain tool compound with nanomolar affinity, to thalidomide, a drug used in the treatment of multiple myeloma. Thalidomide acts by the recruitment of cereblon, a cullin-dependent ubiquitin ligase that marks protein for degradation by the proteasome. The hybrid compounds **72** and **73** hence bind to their bromodomain targets, which then suffer cereblon induced protein degradation. The cellular effects of the hybrids were shown to be more potent and longer lasting than with JQ-1 [126, 127]. The hybrid **73** showed efficacy in a mouse AML xenograft at 50 mg/kg.

## Conclusions

Polypharmacology, rather than a highly specific “magic bullet,” is the norm for small molecule drugs. A recent survey, for example, found that over 40 % of drugs according to the Anatomical Therapeutic Chemical (ATC) classification had a reported IC_50_ < 10 μM for six or more targets [128]. While such promiscuity is usually discovered serendipitiously, it can also be deliberately incorporated. In this review, we have described the two major ways in which this has been achieved within the relatively new area of epigenetic drug discovery. The first is through combination therapy using two independent and relatively selective drugs. At the present time, there are ongoing clinical trials that are combining either an epigenetic and a non-epigenetic drug or two epigenetic drugs with distinct mechanisms of action. In many cases, there is in vitro and in vivo evidence from animal models that such combinations have a synergistic effect. Furthermore, they may help widen the scope of epigenetic drugs beyond the narrow spectrum of hematological cancers for which they are currently approved. The second approach, which is more radical, involves the rational design of a new entity that exerts its biological activity through two or more pathways. In epigenetics, this has been highly successful with HDAC inhibitors due to their simple and tolerant pharmacophore. The literature abounds with examples of multitarget HDAC inhibitors, and in two cases from the company Curis, both linked with dual kinase inhibition, the compounds have completed phase I clinical trials. As our understanding of epigenetic targets and their biological relevance deepens, further progress with epigenetic polypharmacology will certainly be accomplished that directly benefits patients in the clinic.

## Abbreviations

AchE: Acetylcholine esterase
AKT (PKB RAC): Protein kinases B
ALL: Acute lymphoblastic leukemia
AML: Acute myeloid leukemia
APL: Acute promyelocytic leukemia
AR: Androgen receptor
ATC: Anatomical therapeutic chemical
ATRA: All-*trans*-retinoic acid
BCR-ABL: Breakpoint cluster region—Abelson
BET: Bromodomain and extra terminal domain
BIM: B cell chronic lymphocytic leukemia-lymphoma-like 11 gene
BRD: Bromodomain
BRG1: ATP-dependent helicase SMARCA4, a transcription activator
CML: Chronic myelogeneous leukemia
DNMT: DNA methyltransferase
Dot1/DOT1L: Disruptor of telomeric silencing 1
EGFR: Epidermal growth factor receptor
ER: Estrogen receptor
EZH2: Enhancer of zeste homologous 2
FAD: Flavin adenine dinucleotide
GPCR: G protein-coupled receptor
HAT: Histone acetyltransferase
HDAC: Histone deacetylase
HER: Human epidermal growth factor receptor
HKMT: Histone lysine methyltransferase
HMGCR: 3-hydroxy-3-methylglutaryl coenzyme A reductase
IMPDH: Inosine monophosphate dehydrogenase
JHDMs: JmjC domain-containing demethylases
KDR: Kinase insert domain receptor
LSD1/KDM: Lysine specific demethylase 1
MAO: Monoamine oxidase
MDS: Myelodysplastic syndromes
MLL: Mixed lineage leukemia
MM: Multiple myeloma
MTD: Maximum tolerated dose
MW: Molecular weigth
NK-LGL: Natural killer cell large glanural lymphocytes
NMC: NUT midline carcinoma
NSCLC: Non-small cell lung cancer
pAkt: Phospho-protein kinase B
PBMCs: Peripheral blood mononuclear cells
PDE5: Phosphodiesterase 5
pERK: Phospho-protein kinase RNA-like endoplasmatic reticulum kinase
PI3K: Phosphatidylinositol 3-kinase
PML: Promyelocytic leukemia
PR: Progesterone receptor
PRMT: Protein arginine methyltransferase
SAH: *S*-adenosyl homocysteine
SAHA: Suberoylanilide hydroxamic acid
SAM: *S*-adenosylmethionine (*S*-AdoMet)
SERDs: Selective ER downregulator
SERMs: Selective estrogen receptor modulators
SET: Su(var)3-9: suppressor of position-effect variegation; E(z): enhancer of zeste; Trx: trithorax
Sir2: Silent information regulator 2
SIRT: Sirtuins
TCP: Tranylcypromine
TKIs: Tyrosine kinase inhibitors
TSG: Tumor suppressor gene
VEGF: Vascular endothelial growth factor
VEGFR: Vascular endothelial growth factor receptor
